# Families and Social Media Use: The Role of Parents’ Perceptions about Social Media Impact on Family Systems in the Relationship between Family Collective Efficacy and Open Communication

**DOI:** 10.3390/ijerph16245006

**Published:** 2019-12-09

**Authors:** Fortuna Procentese, Flora Gatti, Immacolata Di Napoli

**Affiliations:** Department of Humanities, University of Naples Federico II, 80133 Naples, Italy; flora.gatti@unina.it (F.G.); immacolata.dinapoli@unina.it (I.D.N.)

**Keywords:** social media, family communication, collective family efficacy

## Abstract

Communication through social media characterizes modern lifestyles and relationships, including family interactions. The present study aims at deepening the role that parents’ perceptions about social media effects on family systems can exert within their family functioning, specifically referring to the relationship between collective family efficacy and open communications within family systems with adolescents. A questionnaire to detect the openness of family communications, the collective family efficacy and the perceptions about the impacts of social media on family systems was administered to 227 Italian parents who had one or more teenage children, and who use Facebook and WhatsApp to communicate with them. From the results, these perceptions emerge as a mediator in the relationship between the collective family efficacy and the openness of communications, suggesting that it is not only the actual impact of social media on family systems that matters but also parents’ perceptions about it and how much they feel able to manage their and their children’s social media use without damaging their family relationships. Thus, the need to foster parents’ positive perceptions about social media’s potential impact on their family relationships emerges. A strategy could be the promotion of knowledge on how to functionally use social media.

## 1. Introduction

Families represent not only environments wherein their members live but also whole complex social systems [1,2]. Thus, according to the family systems theory perspective, family functioning refers to processes and interactions in which the members of the system are involved to meet their needs, make decisions, define goals, and establish rules for themselves and for the system as a whole. Levels of openness of communications and healthiness of interactions represent characterizing elements of family’s ability to function adequately, associated with positive outcomes at both individual and family levels [3]. With specific reference to systems including adolescents, mutual acceptance and open communications among family members can help them in managing stressors and negotiating adolescents’ individuation [4], as they allow children to talk with their parents about daily concerns, activities, issues, and in turn, parents being adequately supportive of them [5,6]. 

Moreover, social cognitive theory assigns a central role to perceived efficacy in managing different aspects of daily relationships, interactions, and tasks within the system [7,8]. Specifically, family collective efficacy is “members’ beliefs in the capabilities of their family to work together to promote each other’s development and well-being, maintain beneficial ties to extrafamilial systems, and to exhibit resilience to adversity” ([5], p. 424). Studies [5,9] showed that higher collective family efficacy associates with higher family satisfaction, open communication, effective parental monitoring, and lower aggressive management of conflicts and communication problems. Such an efficacy plays a key role in managing demands and issues related to parenthood [7], representing a protective factor helping parents to get positive outcomes for their family system as a whole.

With reference to family relationships, the most recent literature has deepened the understanding of the impact that social media can have on them with specific attention to particular family tasks, challenges and phases of family life. Social media use can specifically be a central issue for families facing adolescence evolutionary tasks [10,11,12], which also refer to adolescents’ negotiation of autonomy and independence within the family system and to the significance of peer relationships [13]. Indeed, given that nowadays, adolescents spend significant amounts of time using social media with a variety of goals, scholars often talk about Generation M[edia] when referring to modern adolescents [14,15]. This seems to be an increasing trend according to the latest data from the We Are Social report [16], which states that in Italy there are 43.31 million Internet users (10% more than in 2017); 34 million (57%) are active social media users (10% more than in 2017), 30 million (51%) do this through their mobile devices (7% more than in 2017); moreover, 53% of Italian new technology users believe that they offer more risks than opportunities, while 54% state they prefer to use them if it is possible [16]. 

Thus, it is evident that the information and communication technologies (ICTs) are profoundly changing the ways in which people behave and relate to each other [17,18] and creating conflicting perceptions about their impact. As they have become cultural practices embedded in everyday life relationships [3,10,19,20,21,22,23,24,25,26,27], their contribution to creating richer and more complex patterns of interactions [28], including to family life, cannot be ignored [29]; however, whether the effects of these new forms of interactions on the functioning of family systems are positive or negative is still unclear, even more when considering families with adolescents [30]. Thus, with Facebook and WhatsApp being the most used social media in Italy [16], also among relatives, the present study aims at deepening the role that parents’ perceptions about the effects of social media on their family system can exert within the functionality of their family, specifically referring to the relationship between collective family efficacy and open communications within family systems with adolescents.

## 2. Perceptions about Social Media Use within Families with Adolescents

According to Hertlein’s multitheoretical model [31], the ecological influences related to social media features (e.g., accessibility, acceptability, accommodation), the changes social media use brings with reference to family structure (e.g., redefinition of rules, roles, and boundaries), and the ones related to family processes (e.g., redefinition of intimacy, new ways of communicating, new rituals) are interconnected and interdependent. Thus, due to the spread of new ways of communicating and to the consequences they can bring with reference to the functionality and habits of the family (e.g., redefinition of roles and boundaries, new kinds of intimacy, communications, rituals, [29,31]), parents can have ambivalent perceptions about their impact on relationships and communications with their adolescent children. Consistently, studies about families, which include adolescents, brought ambivalent results too, ranging from higher social support [30] to lower family cohesion [31] and progressive isolation of family members within the same house [32,33].

Indeed, on the one hand, ICTs use can provide positive results in terms of family cohesion, adaptability, and open communications [3] and can have a positive impact on family relationships too [34], by allowing family members to keep in touch, make plans in real-time, ensure children’s safety as they allow communications in emergency situations [35], strengthen family ties, encourage parent–child interactions, and promote and facilitate discussions [36]. Moreover, ICTs and social media use could increasingly ensure what Castells [37] defined as autonomy in security conditions, as they help parents in communicating with their children at any time, checking their movements in physical and online spaces [35,38,39,40]. 

On the other hand, the connectedness allowed by mobile devices and social media needs to be negotiated in times, spaces, and occasions where it is allowed, and the chances to perpetually communicate need to be modulated [41]. A risk arising from the lack of modulation and negotiation about social media and mobile devices use, which could impact family relationships and dynamics, seems related to the phubbing phenomenon, i.e., ignoring someone in a social environment by paying attention to mobile devices instead (e.g., interrupting a meal while eating together to check the phone for messages or missed calls) [42,43]. Altogether, the arrangements needed to avoid these kinds of risks and modulating mobile devices use in times, spaces, and occasions could cause conflicts within families [35,39,41,44,45,46], as parents who are more worried about social media impacts can exert a greater control over their children’s use [47,48,49], making adolescents get the perception of being hyper-controlled by their parents, that in turn can increase the level of conflict and aggressive communications. Moreover, as social media represents environments wherein different social norms and rules can be established and followed by adolescents out of their parents’ control, this can make further risks arise if their use and its consequences is not adequately discussed among family members, as, therefore, adolescents’ decision-making processes can be affected by those norms (e.g., [50]).

## 3. Aim of the Study

It has been acknowledged that the perceived collective family efficacy refers to the perception about family members being able to handle daily social interactions, challenges, and communications within the system and helps in achieving positive family outcomes such as open communications [5]. Thus, as the widespread ITCs use within families represents a new challenge to be managed by parents through an active adaptation, which can bring changes in family communications [30,31] and habits, beliefs and norms [29], the following hypothesis is suggested:

H: Parents’ perception of the impact of social media use on their family system mediates the relationship between their perceived collective family efficacy and the perceived openness of communications within the family system.

Open communication has been chosen as a key outcome because it can be a particularly relevant issue in family systems which include adolescents [51,52]. 

## 4. Materials and Methods

### 4.1. Participants and Procedures

Snowball sampling was used to recruit 227 Italian parents with one or more teenage child (aged between 13 and 19), who use Facebook and WhatsApp to communicate with them; having at least one teenage child and communicating with s/he through smartphones and ICT was the criterion to be a participant in the study. The researchers paid attention to privacy and ethics, and introduced the questionnaire with an explanation about confidentiality and anonymity issues, conforming with the International applicable law (EU Reg. 2016/679). At the end of this explanation, every participant had to express his/her informed consent; in case of a negative answer, they could not take part in the study. They received no compensation for participating in the study. 

Seventy percent were female, 30% male; 25.1% were born between 1943 and 1960 (the so-called “Baby Boomers”, [53]), 68.3% between 1961 and 1981 (the so-called “Gen Xers”, [53]), 6.6% between 1982 and 1997 (the so-called “Millennials”, [53]); 11.5% were from Northern Italy, 8.4% from Central Italy and 77.5% from Southern Italy; only 2.6% were from Italian islands. Most of the participants (72.2%) were married or cohabiting, while 15.9% were separated or divorced, 8.8% unmarried, and 3.1% widower. About half the participants (48.9%) had a high school diploma, while 26% a Bachelor’s or Master’s degree and 7.9% a higher degree; 14.1% had a secondary school diploma.

### 4.2. Measures

The questionnaire included a section about socio-demographic data and the following measures.

#### 4.2.1. Collective Family Efficacy

The collective family efficacy scale (α = 0.96, [8]) was used. It is compounded by 20 items on a 7-point Likert scale (1 = Not well at all; 7 = Very well), aimed at measuring the perceived operative capabilities of the family as a whole system, such as managing daily routines, achieving consensus in decision making and planning, coping together with adversities, promoting reciprocal commitment, providing emotional support when needed, enjoying the time together. Being interested in the holistic efficacy appraisal [54,55], the total score was used.

#### 4.2.2. Family Open Communication 

A pool of 8 items (α = 0.90, see Table 1 for the items) was used to detect participants’ perceptions about the openness of their family communications. Respondents were asked to rate their level of agreement with each item on a 5-point Likert scale (1 = Strongly disagree; 5 = Strongly agree). 

#### 4.2.3. Social Media Impact on Family Systems. 

A pool of 9 items (α = 0.73, see Table 2 for the items), referring to both positive and negative impacts of social media on family systems, was used to assess participants’ perceptions about it. Respondents were asked to rate their agreement with each item on a 5-point Likert scale (1 = Strongly disagree; 5 = Strongly agree). As positive and negative impacts of social media use on family systems can be meant as two sides of the same coin, the total score was used.

### 4.3. Data Analysis

#### 4.3.1. Preliminary Analyses

As they had not been validated yet, exploratory factor analyses (EFA) with principal axis factoring and promax rotation were led to extract the factors of the family open communication and of the social media impact on family system scales. For both scales, sphericity was checked using Bartlett’s test and adequacy of sampling using the Keiser Meyer Olkin (KMO) measure. The emerged factor structures were further tested through confirmatory factor analyses (CFA) run with structural equation modeling (SEM). Specifically, for the social media impact on family system scale a two-factor structure, as suggested by the EFA, and a hierarchical structure with the two factors loading on a higher-order latent dimension were tested to determine which one better fitted the data, consistently with the theoretical model about positive and negative impacts of social media use on family system as two sides of the same coin.

For the family collective efficacy, the factor structure that emerged from a previous study [8] was tested through CFA run with SEM. 

To evaluate the model fit for all the CFA, different indices were observed [56]: The Chi-square test of model fit, the comparative fit index (CFI), the standardized root mean square residual (SRMR). For the CFI, values equal to or greater than 0.90 e 0.95 reflect good or excellent fit indices, respectively; for the SRMR, values equal to or smaller than 0.06 e 0.08 reflect good or reasonable fit indices, respectively [57]. Moreover, when it came to testing which model better fitted the data for the social media impact on the family system scale, the Akaike information criterion (AIC) and the Bayesian information criterion (BIC) were also used; for both indices, the lower the value, the better the fit.

#### 4.3.2. Hypothesis Testing

The mediation hypothesis was tested through SEM. Collective family efficacy was the independent variable, openness of family communications was the dependent one; the perception about social media impact on family systems was the mediator; participants’ age and sex were modeled as covariates on all the variables in the model. A dummy variable was created for participants’ sex before entering it in the model (0 = male / 1 = female).

Before testing the hypothesis, the presence of outliers and/or influential cases was checked using the leverage value and Cook’s D to test the absence of significant values in the data affecting the analyses [58]. Multicollinearity was tested through condition and tolerance indexes [59]. Common variance was controlled through Harman’s single-factor test [60].

Given the interest in higher-order constructs, a heterogeneous parceling was adopted [61], as it reproduces smaller but more reliable coefficients than the homogeneous one [62] and allows for creating parcels without generating a flawed measurement model because theoretically meaningful categories were included in the SEM. 

To evaluate the model fit, the following indices of fit were observed [56]: The Chi-square test of model fit, the CFI, the SRMR. 

Bootstrap estimation was used to test the significance of the results [63,64] with 10,000 samples, and the bias-corrected 95% CI was computed by determining the effects at the 2.5th and 97.5th percentiles; the indirect effects are significant when there is no 0 in the CI.

## 5. Results

For the family open communication scale and for the social media impact on family system scale, sphericity (family open communication scale: Chi-square (28) = 974.765, *p* < 0.001; social media impact on family system scale: Chi-square (36) = 756.527, *p* < 0.001) and adequacy of sampling (0.893 for the family open communication scale, 0.747 for the perceptions about social media impact on family system scale) reported good values. No item was deleted from the original pools due to too low loadings nor too high loadings on more than one factor; all the items in the final versions of the scales had loadings above 0.3 in only one factor (see Table 1 and Table 2).

The CFA confirmed an adequate model fit for the family open communication scale, Chi-square (19) = 105.100, *p* < 0.001, CFI = 0.91, SRMR = 0.05, and for the family collective efficacy scale, Chi-square (169) = 789.980, *p* < 0.001, CFI = 0.94, SRMR = 0.02. For the social media impact on family system scale, the hierarchical model, Chi-square (22) = 98.878, *p* < 0.001, CFI = 0.90, SRMR = 0.06, AIC = 5714.096, BIC = 5816.844, better fitted the data than the two-factor model, Chi-square (24) = 98.878, *p* < 0.001, CFI = 0.89, SRMR = 0.07, AIC = 5718.096, BIC = 5827.694, confirming positive and negative impacts of social media use as two sides of the same coin. 

The descriptive statistics and the correlations for all the measures are in Table 3.

### Hypotheses Testing

Since the leverage value was always lower than 0.09 and Cook’s D lowest and highest values were 0 and 0.36, there were no significant values in the data affecting the analyses; as the variables in the model had Tolerance indexes between 0.88 and 0.98, multicollinearity among them was not a problem [59].

**Figure 1:**
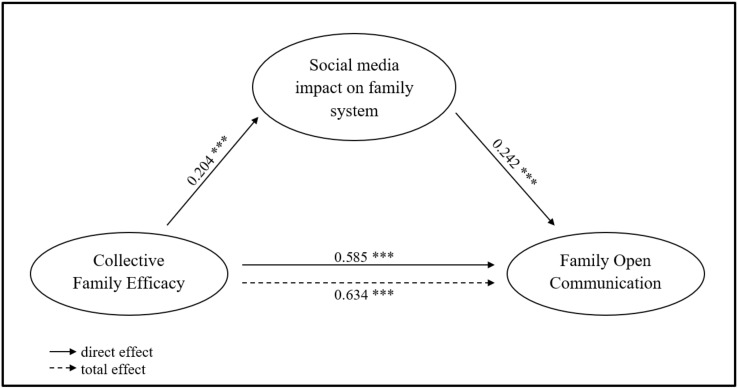
Mediation model. Note. *n* = 227. *** *p* < 0.001. Unstandardized coefficients (*B*) are shown.

The hypothesized mediation model (see Figure 1) showed good fit indices, Chi-square (33) = 50.280, *p* < 0.027, CFI = 0.99, SRMR = 0.02. 

Collective family efficacy emerged as a significant predictor of the openness of family communications, *B* = 0.585, S.E. = 0.07, *p* < 0.001, bias-corrected 95% CI [0.410, 0.709], and of the perceptions about social media impact on family systems, *B* = 0.204, S.E. = 0.064, *p* = 0.001, bias-corrected 95% CI [0.067, 0.321]; the latter was a significant predictor of the openness of family communications too, *B* = 0.242, S.E. = 0.056, *p* < 0.001, bias-corrected 95% CI [0.126, 0.342]. The indirect effect of collective family efficacy on openness of family communications via the perceptions about social media impact on family systems was small yet significant, *B* = 0.049, S.E. = 0.019, *p* = 0.01, bias-corrected 95% CI [0.02, 0.098], supporting the hypothesis of partial mediation. The unstandardized total effect was 0.634, S.E. = 0.066, *p* < 0.001, bias-corrected 95% CI [0.459, 0.747]. 

Participants’ sex emerged as a significant predictor only for the perceptions about social media impact on family systems, *B* = -0.132, S.E. = 0.105, *p* = 0.008, bias-corrected 95% CI [−0.433, −0.022]; participants’ age was significant only for the collective family efficacy, *B* = 0.081, S.E. = 0.072, *p* = 0.05, bias-corrected 95% CI [0.004, 0.279].

## 6. Discussion

The present study deepens the understanding of how social media can produce changes within family systems, taking into consideration the role that parents’ perceptions about the impact of social media on family systems, whether positive or negative, can exert in the relationship between their perceived collective family efficacy and an open communication among family members; specifically, the leading hypothesis referred to the mediator role of these perceptions, whether positive or negative, in the above-mentioned relationship. The results confirm the hypothesis, showing that parents’ perceptions represent a partial mediator of the relationship between their perceptions about collective family efficacy and openness of communications; nevertheless, the indirect effect of collective family efficacy on openness of family communications via parents’ perceptions about the impact of social media on family systems was small, showing that all the direct effects in the model were still bigger.

It has already been widely acknowledged that social media and ICTs make human social interactions and relationships more complex; however, scientific results still showed conflicting results about whether such complexity can have a positive, enriching, role or rather than a negative, detrimental, one with reference to family interactions, even more when the family system includes adolescent children [36]—due to the evolutionary tasks they have to face up to, which can impact on family relationships and interactions temporarily or permanently [10,11,12,13]. These results provide further hints about social media role within family relationships and functioning.

Indeed, while it is well established that family collective efficacy can have a boosting role with reference to healthy interactions and open communications within the family system [5,10], what emerged here suggests that it is not only the real impact of social media on family systems [36] that matters but also how family members perceive it and how much they feel confident about their family managing daily challenges to achieve positive relationships, healthy interactions, and open communications. Indeed, the results show that that being confident in one’s family capabilities to handle daily tasks, stress, and challenges associates with a more positive perception about the impact social media can have on family system and the relationships within it, as feeling able to manage family daily tasks and challenges could foster the feeling about being able to manage the adaptation to the increasing social media use among family members too. This could make family members perceive, at last, these new technologies as opportunities for increased family cohesion, adaptability, interactions, planning, and open communications [3,34,36], rather than as threats to positive family functioning and relationships. In addition to family collective efficacy, also such positive perception can further promote open communications among family members, maybe because if social media are perceived as opportunities and useful tools they can offer further ways to maintain and improve relationships among family members (e.g., to keep in touch, make plans in real-time, promote and facilitate discussions, and encourage parent–child interactions, [35,36]). When parents are aware of their family’s ability to manage social media-related changes in family functioning and habits (e.g., redefinition of roles and boundaries, new kinds of intimacy, communications, rituals, [29,31]), this can foster their perception about potentialities and new opportunities coming from social media use to keep in touch with their children, most of all when they are adolescents and are facing up their individualization process: if parents are able not to make their children feel they are invading their privacy or being oppressive and hyper-controlling, and to discuss with them how social media should be used to reduce the risks, social media can at last strengthen family ties, promote and facilitate discussions, and foster more secure conditions for adolescents to obtain greater autonomy from their parents and for parents to let them face up to these situations [35,36,38,39]. Indeed, when used in a responsible and aware way, social media can represent a resource and an educational added value within family relationships, helping parents to exploit a new educational and participative space that could strengthen the relationships with their children. This seems also consistent with previous results about how social media use can enhance the opportunities for a more open dialogue between parents and children, allowing the latter to get closer to the language and lifestyles of the first ones and to share with them important, sensitive and/or educational discussion topics functional to their growth [36]. Thus, social media may foster open communications among family members and a supportive family environment wherein adolescents can grow up and face up to their evolutionary tasks and subsequent stressful events, getting positive outcomes [36]. 

## 7. Conclusions

The study shows the relevance that parents’ positive perceptions about the impact of social media on social interactions and relationships within their family system can have in fostering a good family functioning and open communications among family members. Moreover, with reference to the role that collective family efficacy exerts, it also suggests that relying on family abilities to manage daily life tasks and face daily challenges could represent a strategy to promote the acknowledgment that challenges related to social media uses, their consequences, and the potential subsequent risks could be managed with adequate information and negotiation of the changes they bring in terms of family communications, habits, interactions, and rituals among parents. Taking into consideration the results from this study, an emergent issue seems related to the need to promote a wider acknowledgment that social media can be positively and functionally used among modern parents [36], showing them different ways in which social media can represent educational and participative spaces aimed at promoting a wider and more open communication between them and their children and a critical and responsible awareness for their children at the same time, fostering, at last, their positive perceptions about social media impact on family systems. Indeed, social media accessibility, acceptability, and accommodation require the redefinition of rules and roles, producing new processes and dynamics within family systems [31] parents have to deal with if they want to get a positive perception about their use: if adequately managed, these processes can allow the creation of further spaces wherein the relational dynamics between parents and adolescent children can happen and be successfully managed. Consistently, the aspects that emerged from this study invite to set up further studies aimed at deepening the meaning that social media tools can assume in the construction of transition spaces, allowing the expression and mediation of the divergences and conflicts that can show up in families with adolescent children.

It is important to also acknowledge some limitations of this study. 

First, it takes into consideration only the parents’ perspective, but a major comprehension of family relationships should take into consideration the children’s perspective also, or even a dyadic one. Moreover, the findings are based on self-reported data, which can become distorted due to problems related to memory bias and response fatigue. 

Lastly, another issue refers to the cross-sectional design of the study; thus, the relationships described should be considered carefully, and no causal inference is possible. 

It would also be useful to extend the analyses to samples from other countries, to verify whether and how the cultural and community [65,66,67,68] dimensions modify the perceptions about social media impact within family systems and their effect on family communications.

## Figures and Tables

**Table 1:** Factor loadings for exploratory factor analyses (EFA) with principal axis factoring for the family open communication scale.

Item	Factor Loading
Every member of my family is satisfied about how we communicate.	0.696
Each one among us listens to the other members of the family.	0.813
Each one among us knows how to express love to the other members of the family.	0.767
Each one among us can ask whatever s/he wants to the other members of the family.	0.698
Each one among us can talk about his/her problems with the other members of the family.	0.722
Each one among us can talk about his/her ideas and beliefs with the other members of the family.	0.796
Each one among us tries to understand other members’ feelings.	0.722
Each one among us expresses whatever s/he feels to the other members of the family.	0.640
Explained variance (%)	53.846
Cronbach’s α	0.90

Note. *n* = 227.

**Table 2:** Factor loadings for EFA with principal axis factoring and promax rotation for the social media impact on the family system scale.

Item	Positive Impact	Negative Impact
They improve a healthy communication.	0.715	
They interfere with family rules. *		0.554
They improve family cohesion.	0.789	
They expose family privacy to risks. *		0.712
They help in bounding generations.	0.723	
They expose family intimacy to risks. *		0.785
They help in facing up to life cycle transitions.	0.678	
They make the relationships among family members more vulnerable. *		0.699
They strengthen family resilience (that is the ability to face up positively to traumatic events, to reorganize functionally after some difficulties).	0.715	
Explained variance (%)	29.306	21.527
Cronbach’s α	0.73	

Note. *n* = 227 * item score is reversed. Only factor loading > 0.30 are shown.

**Table 3:** Descriptive statistics and correlations.

Variables	Range	*M*	*SD*	1	2
1. Collective family efficacy	1–7	4.98	1.04	-	
2. Family open communication	1–5	3.72	0.79	0.509 ***	-
3. Social media impact on family system	1–5	2.87	0.64	0.140 *	0.122

Note. *n* = 227. *** *p* < 0.001 (2-tailed); * *p* < 0.05 (2-tailed).
